# A mesoporous electrode strategy for probing ions at the electrode–electrolyte interface using *in situ* X-ray absorption spectroscopy

**DOI:** 10.1039/d6cp01946c

**Published:** 2026-10-27

**Authors:** Jan Sebastian Dominic Rodriguez, Alfred Larsson, Nipon Deka, Rianne Jorritsma, Hassan Javed, Kees Kolmeijer, Ajsa Cuprija, Wanli Liu, Nicole Barber, Adam M. Squires, Detre Teschner, Aliakbar Ghafari, Cheng-Hao Chuang, Rik V. Mom

**Affiliations:** a Leiden Institute of Chemistry, Leiden University Leiden The Netherlands r.v.mom@lic.leidenuniv.nl; b Department of Physics, Tamkang University New Taipei City Taiwan; c Department of Atomic-Scale Dynamics in Light-Energy Conversion, Helmholtz-Zentrum Berlin für Materialien und Energie Albert-Einstein-Straße 15 12489 Berlin Germany; d Division of Synchrotron Radiation, Lund University Lund Sweden; e Department of Chemistry, University of Bath Bath BA2 7AY UK; f Department of Inorganic Chemistry, Fritz-Haber-Institut der Max-Planck-Gesellschaft Faradayweg 4–6 14195 Berlin Germany; g Max Planck Institute for Chemical Energy Conversion Stiftstrasse 34-36 45470 Mülheim an der Ruhr Germany

## Abstract

Electrolyte ions present at the electrode–electrolyte interface critically influence electrocatalytic reactions, yet direct, quantitative detection of interfacial ions under operating conditions remains challenging due to dominant bulk electrolyte signals. Here, we present an interface-sensitive soft X-ray absorption spectroscopy (XAS) approach that enables quantitative tracking of interfacial ion behavior at electrified platinum (Pt) surfaces. Mesoporous Pt films with high surface-area-to-volume ratios were fabricated *via* soft-templated electrodeposition onto SiN_*x*_ windows, strongly enhancing the relative contribution of the interfacial electrolyte within nanoscale pores while effectively attenuating signals from the bulk. Using Na^+^ as a model cation, Na K-edge XAS measurements reveal pronounced dependencies of interfacial ion concentration on applied potential, electrolyte pH, and bulk concentration. The observed trends highlight the role of surface chemistry in interfacial ion behavior. In addition to concentration changes, potential-dependent modifications of the Na^+^ hydration shell are identified, indicating re-organization of the solvation of the ions at the interface. These results highlight the capability of our methodology to quantitatively probe ion-specific behavior in the electrical double layer under *operando* conditions. We foresee that the method can be extended to a wide range of ionic and molecular species relevant to electrocatalysis and energy conversion.

## Abstract 2

Electrolyte ions at the interface critically influence electrocatalysis, yet their direct, quantitative detection during operating conditions remains challenging due to dominant bulk electrolyte signals.

## Graphical abstract

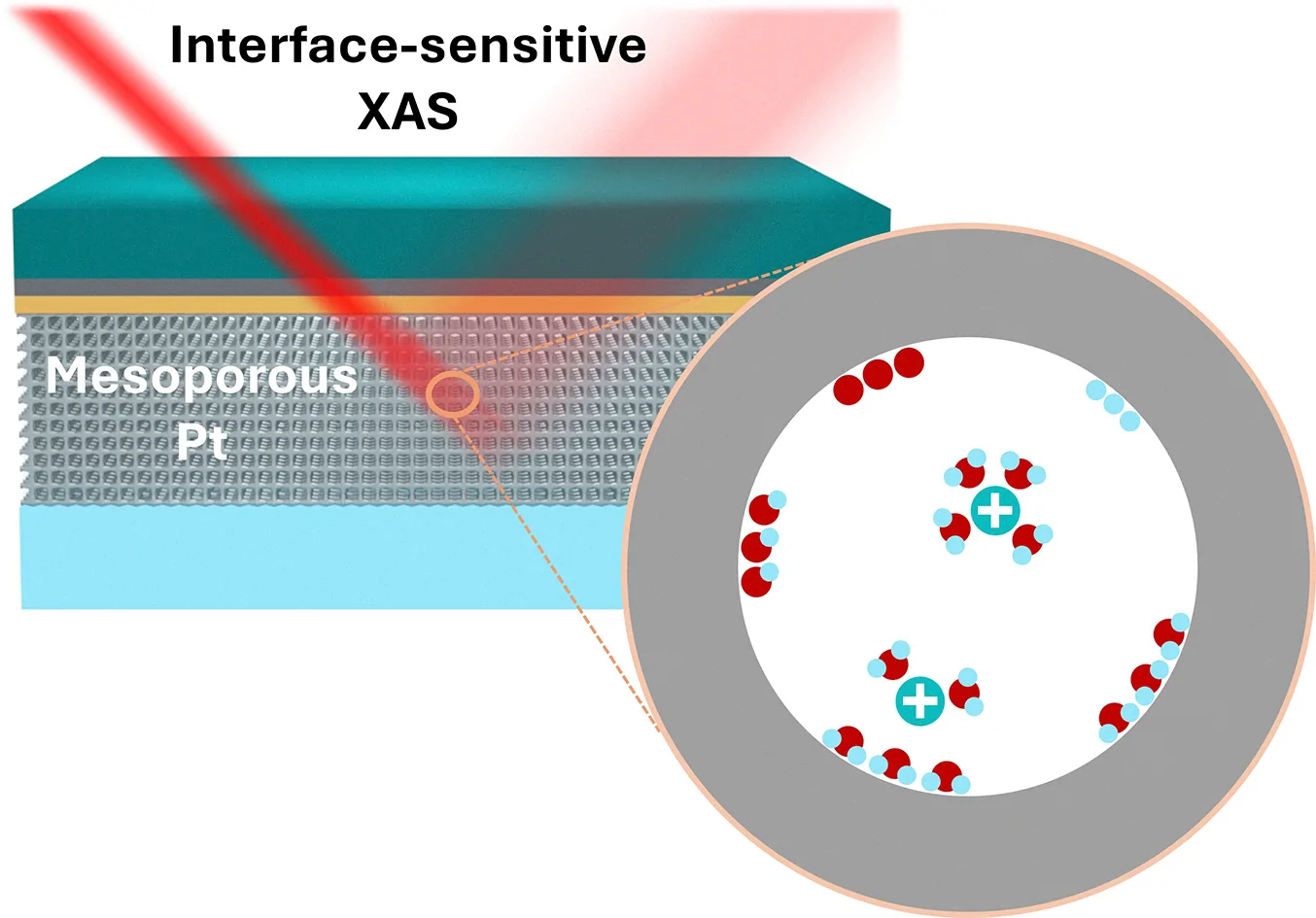

## Introduction

For electrochemical reactions such as the hydrogen evolution (HER), oxygen evolution (OER), and oxygen reduction (ORR), ions present at the electrode–electrolyte interface have a strong impact on the catalytic activity.^1–5^ Exploiting this, the type and concentration of ions in the electrolyte, and the electrolyte's pH have all been used to tune electrocatalytic performance. These effects stem from the interactions of water molecules, ions, and adsorbates at the electrode–electrolyte interface. However, the detailed structure of this interface, and how it changes with potential and surface chemistry, is still not fully understood.

In particular, the behavior of cations near the electrode surface has proven challenging to resolve. In the bulk electrolyte, cations are solvated and well-distributed. However, at the charged electrode surface, cations can be attracted or repelled and may be either solvated or partially desolvated, depending on the applied conditions.^6–8^ This interfacial ion behavior has a significant impact on electrocatalysis, as the interfacial ion structure tunes the catalytic reaction environment by changing the local electric field, modifying the interfacial water structure, or by stabilizing intermediates through ion–adsorbate interactions.^1,2,5^ Therefore, it is important to quantify how many cations are located near the surface during reaction conditions.

A main experimental challenge to detect interfacial cations is that the electrode–electrolyte interface, where they are located, is only a very small part of the whole electrochemical cell. For most characterization techniques, the signal primarily comes from the bulk electrolyte, making it difficult to isolate signals from the interface. To solve this, several interface-sensitive techniques have been developed, which use specific types of light-matter interactions to induce interface sensitivity.^9^ For example, surface X-ray diffraction (XRD) exploits total reflection,^10–12^ sum-frequency generation (SFG) spectroscopy uses interface-specific selection rules,^13–15^ and plasmon-based methods such as surface-enhanced Raman spectroscopy (SERS) and surface-enhanced infrared spectroscopy (IRAS) enhance signals locally through electronic resonance.^4,11,16^ While each of these strategies has successfully provided insight into interfacial ion behavior, they do have limitations in the types of ions and electrodes that can be studied. Furthermore, it is often difficult to quantify the interfacial ion concentration from the spectra for a series of potentials and electrolytes, which is crucial to relate interfacial ion behavior to catalytic performance trends.

Here, we present an interface-sensitive X-ray absorption spectroscopy approach that enables such quantitative tracking of interfacial ion behavior. We use mesoporous Pt electrodes prepared by soft templating to increase the amount of electrode–electrolyte interface, thereby strongly favoring interfacial ion signals over the bulk signal in (soft) X-ray absorption spectroscopy (XAS). We exemplify this with the case study of Na^+^–Pt interactions, showing how both the applied potential and electrolyte pH strongly impact the interfacial Na^+^ concentration.

## Methods

### Mesoporous electrode preparation

To obtain electrodes that are suitable for interface-sensitive XAS, mesoporous Pt films were electrodeposited on a 20 nm Au/SiN_*x*_ chip (Silson, 100 nm 1 × 1 mm^2^ window) *via* a soft templating process.^17–19^ Prior to the deposition, the SiN_*x*_ chip was cleaned by soaking in a 0.5 M H_2_SO_4_, 1 g L^−1^ KMnO_4_ solution, followed by soaking in a dilute Piranha solution and rinsing with ultrapure water. The plating solution was prepared by dissolving 0.125 g of Brij 58 surfactant and 1.2 mL of 8 wt% hexachloroplatinic acid (HCPA) in 10.77 mL of ultrapure water. The SiN_*x*_ chip was submerged into a glass beaker containing the plating solution, except for a small corner used to make electrical contact. The reference and counter electrodes of the deposition cell were an Ag/AgCl electrode (Innovative Instruments LF-1.6-48 leak-free Ag/AgCl) and a Pt wire, respectively. Deposition of Pt was carried out at 0.27 V_RHE_ unless stated otherwise. As the SiN_*x*_ chip itself is electrically insulating, electrodeposition only occurred on the Au-coated part of the chip. After electrodeposition, the surfactant template was removed by repeated soaking and washing in ethanol and 1 : 1 ethanol–water mixtures, leaving behind a Pt film with mesopores.

### Electrode characterization

The electrochemically active surface area (ECSA) of the mesoporous Pt films was determined using the hydrogen underpotential deposition (H_upd_) charge in 0.1 M HClO_4_, employing the known H_upd_ charge of 210 uC cm^−2^ as conversion factor.^20–22^ Assessment of the film thickness and morphology were carried out using scanning electron microscopy (SEM) using a Zeiss EVO microscope. Further structural characterization was performed using a Anton Paar SAXSpoint 2.0 setup was operated using a Cu source beam (1.54 Å, 8 keV) with detector distance of 350 mm, to obtain small angle X-ray scattering (SAXS) 1-D profiles.

### *In situ* X-ray absorption measurements

The *in situ* XAS measurements were performed at the ISISS beamline endstation at the BESSY II synchrotron facility in Berlin, Germany, which provides monochromatized X-ray photon beams in the soft X-ray regime (80–2000 eV) using a bending magnet (D41) and a plane grating monochromator (PGM). The total fluorescence yield (TFY) signal was recorded using photodiode detectors, with incidence and exit angles of 57° and 55° *vs.* sample normal, respectively. Since the recorded spectra were measured at the Na K-edge, the analysis chamber was kept under high vacuum conditions.

The spectro-electrochemical flow cell features a Pt wire as the counter electrode and an Ag/AgCl electrode (Innovative Instruments LF-1.6-48 leak-free Ag/AgCl) as the reference electrode. The cell can accommodate 1 × 1 cm^2^-frame SiN_*x*_ chips. In this study, we used chips with Au coating and a 1 × 1 mm^2^ 100 nm window (Silson). Electrolyte circulation was achieved by delivering Ar purged electrolyte solutions into the flow cell using a peristaltic pump. Electrochemical control was provided by an SP-200 BioLogic potentiostat, enabling the application of standard electrochemical techniques, including cyclic voltammetry (CV) and chronoamperometry during *operando* spectroscopic measurements.

Prior and during the measurements, several checks were regularly performed to verify the proper wetting, cleanliness, stability and electrochemical behavior of the system. These are described in detail in SI Section S2. A detailed description of the XAS data treatment is provided in SI Section S3.

## Results

Fig. 1 displays the experimental geometry of the interface-sensitive XAS experiments. The basis of the experiment is the SiN_*x*_ window type flow cell that is available at several beamlines,^23–30^ in which a SiN_*x*_ window provides a high-vacuum leaktight separation between the liquid environment in the cell and the vacuum of the spectroscopy chamber. The mesoporous Pt film was coated onto the SiN_*x*_ window, serving as the working electrode. In this geometry, incident X-rays entering the cell first travel through the mesoporous film before they reach other parts of the cell. If the Pt film is sufficiently thick, the X-ray beam is fully scattered and absorbed within the mesoporous film, so that only the mesoporous film and the electrolyte inside the pores are probed in the XAS measurements. Due to the mesoporous structure of the film, a large fraction of this in-pore electrolyte is in contact with the Pt electrode. As a result, the (fluorescence yield) XAS signal from the absorption edges of the ions in the pores is strongly interface-sensitive. In the following, we will show that this makes it possible to detect subtle changes in interfacial ion concentration and solvation structure.

**Fig. 1:**
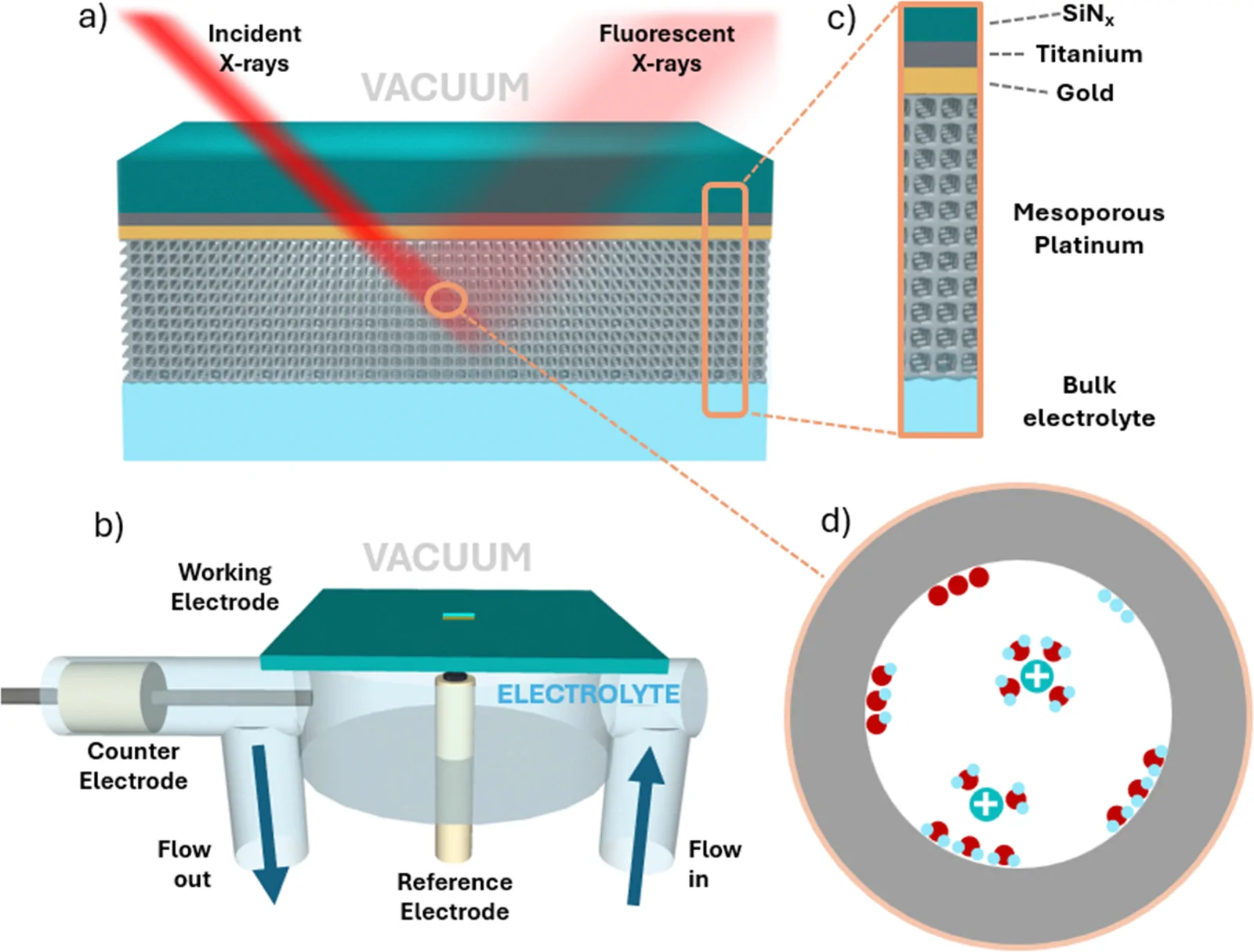
Schematic diagram of the interface-sensitive XAS approach to probe the electrode–electrolyte interface. (a) Sample and (b) liquid flow cell geometries, where the working electrode is composed of (c) a mesoporous Pt film deposited on an Au-coated SiN_*x*_ window with a Ti seed layer. (d) Schematic diagram of the possible adsorbate and electrolyte structures inside the pores.

A key factor in our measurement strategy is that only the electrolyte in the pores should be probed, and not the bulk electrolyte behind the mesoporous film. To this end, the mesoporous film thickness should be chosen such that X-rays are traveling to and from the bulk electrolyte are (almost) fully attenuated. The relative contribution of pore and bulk ion signal can be calculated using:1
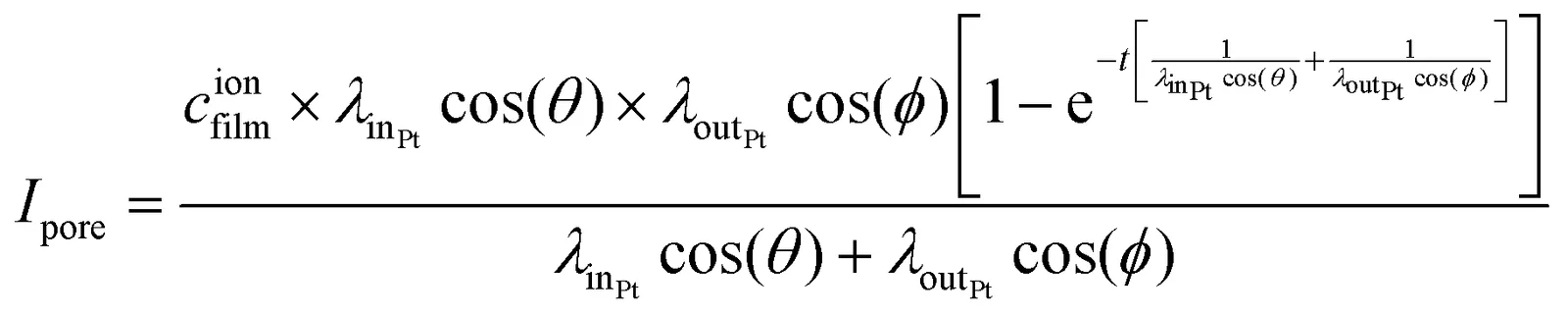
2
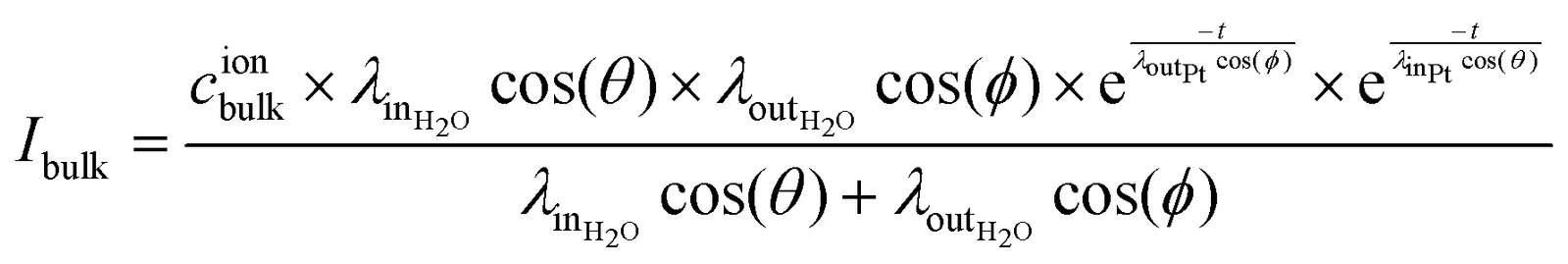
where *I*_pore_ and *I*_bulk_ are the fluorescence signal intensities from ions inside the pores, and in the bulk, respectively. *λ*_in_Pt__ and *λ*_out_Pt__ are the attenuation lengths of the incident and the emitted X-rays inside the mesoporous Pt catalyst, respectively. 
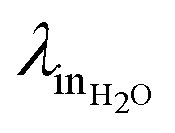
 and 
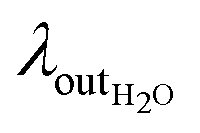
 are the attenuation lengths of the incident and the emitted X-rays within the bulk, respectively. Values for the attenuation lengths of both the mesoporous film and bulk electrolyte can be calculated as described in SI Section S1, exploiting the CXRO database.^31^*c*^ion^_film_ and *c*^ion^_bulk_ are the concentrations of electrolyte ions within and outside the pores of the film, respectively. Finally, *ϑ* and *ϕ* are the X-ray incidence angle and fluorescence collection angle, respectively.

The calculated fluorescence contribution from within the pores increases with film thickness, approaching unity once the majority of the excitation and emitted photons originate from within the porous layer. As shown in Fig. 2a, variations in incident and exit angles slightly shift the thickness required to reach full pore contribution, reflecting changes in the effective X-ray path length through the film. In contrast, Fig. 2b demonstrates a stronger dependence on the attenuation lengths of different electrolyte ions. Species with lower energy edges (*e.g.* Na), and therefore shorter X-ray attenuation lengths, reach near-complete pore contribution at smaller porous film thicknesses, while ions with longer attenuation lengths (*e.g.* K) require substantially thicker films before the fluorescence signal becomes dominated by the pore volume.

**Fig. 2:**
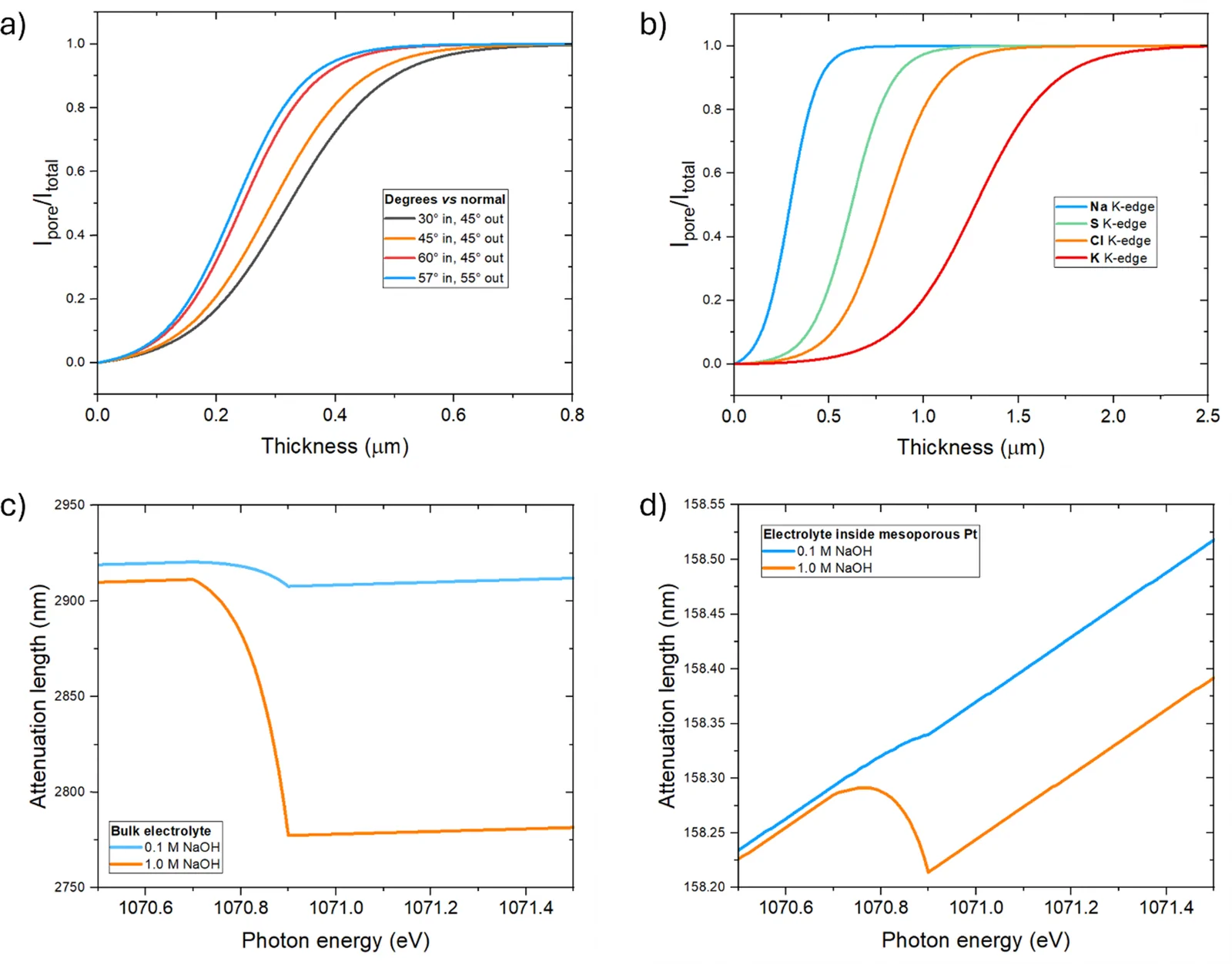
Ion signal contribution from inside the pores for varying mesoporous Pt film thickness. (a) Calculated pore signal contribution, *I*_pore_/*I*_total_, as a function of catalyst film thickness for different incident and exit angles relative to the surface normal. (b) Calculated pore signal contribution for representative commonly used electrolyte ions (Na, S, Cl, and K) at their respective K-edges. X-ray attenuation length around the Na K-edge of (c) bulk NaOH electrolyte solutions and (d) a mesoporous Pt layer with NaOH within the pores at varying concentrations.

For quantitative analysis of the interfacial ion concentration, it is important to ensure that there is a linear relationship between the ion concentration in the pores and the XAS intensity. This condition is met if the ions do not contribute significantly to the overall X-ray attenuation (*i.e.* there is essentially no self-absorption). As shown in Fig. 2c, in bulk electrolyte (*i.e.* without the presence of the mesoporous Pt), there is a clear effect on the attenuation length for concentrated electrolytes (∼4.5% change in attenuation length across the Na K-edge), whereas the effect is minimal in dilute solutions (∼0.3% change in attenuation length). Within the mesoporous Pt film, the effect of X-ray absorption by Na^+^ on the attenuation length is much smaller, because the Pt film dominates the overall X-ray attenuation (see Fig. 2d). Even at a high in-pore ion concentration of 1 M, the X-ray attenuation length hardly changes across the Na K-edge. Therefore, self-absorption effects are expected to play a role only for extremely concentrated electrolytes (*e.g.* water-in-salt electrolytes or ionic liquids).

As discussed above, the preparation of mesoporous Pt films with controlled thickness and pore size is crucial to facilitate XAS measurements on interfacial ions. Such controlled synthesis is possible using soft-templated Pt electrodeposition. In this methodology, self-assembling surfactant structures such as micelles are used to create nanometric voids during Pt electrodeposition (Fig. 3a). Here, we used a robust and straightforward one-pot synthesis process with Brij-58 micelles as the template.^18,32^ The amount of Brij-58 that was added in a dilute solution of HCPA is just slightly above its critical micelle concentration (∼0.8 wt.%) to ensure formation of independent micelles. Under these conditions, Pt^4+^ precursor species preferentially associate with the outer shell of the micelles, where the hydrophilic heads of the Brij-58 chains are located.^18^ As electroreduction proceeds, Pt nucleates at the electrode surface and grows around the surfactant assemblies, forming a continuous metallic framework. Subsequent removal of the surfactant yields a mesoporous Pt structure with a well-defined pore structure, as inferred from the pronounced peak in the 1-D SAXS profile in Fig. 3b. The peak maximum at ∼0.82 nm^−1^ implies a dominant pore-to-pore distance of ∼7.7 nm. At lower *q* values, no further features are observed, indicating that the pore network is disordered. However, at the larger scale, it can be observed that the film grows extremely uniform (Fig. 3c and d). In the context of the interface-sensitive XAS this is highly relevant, as it means that a well-defined film thickness can be chosen.

**Fig. 3:**
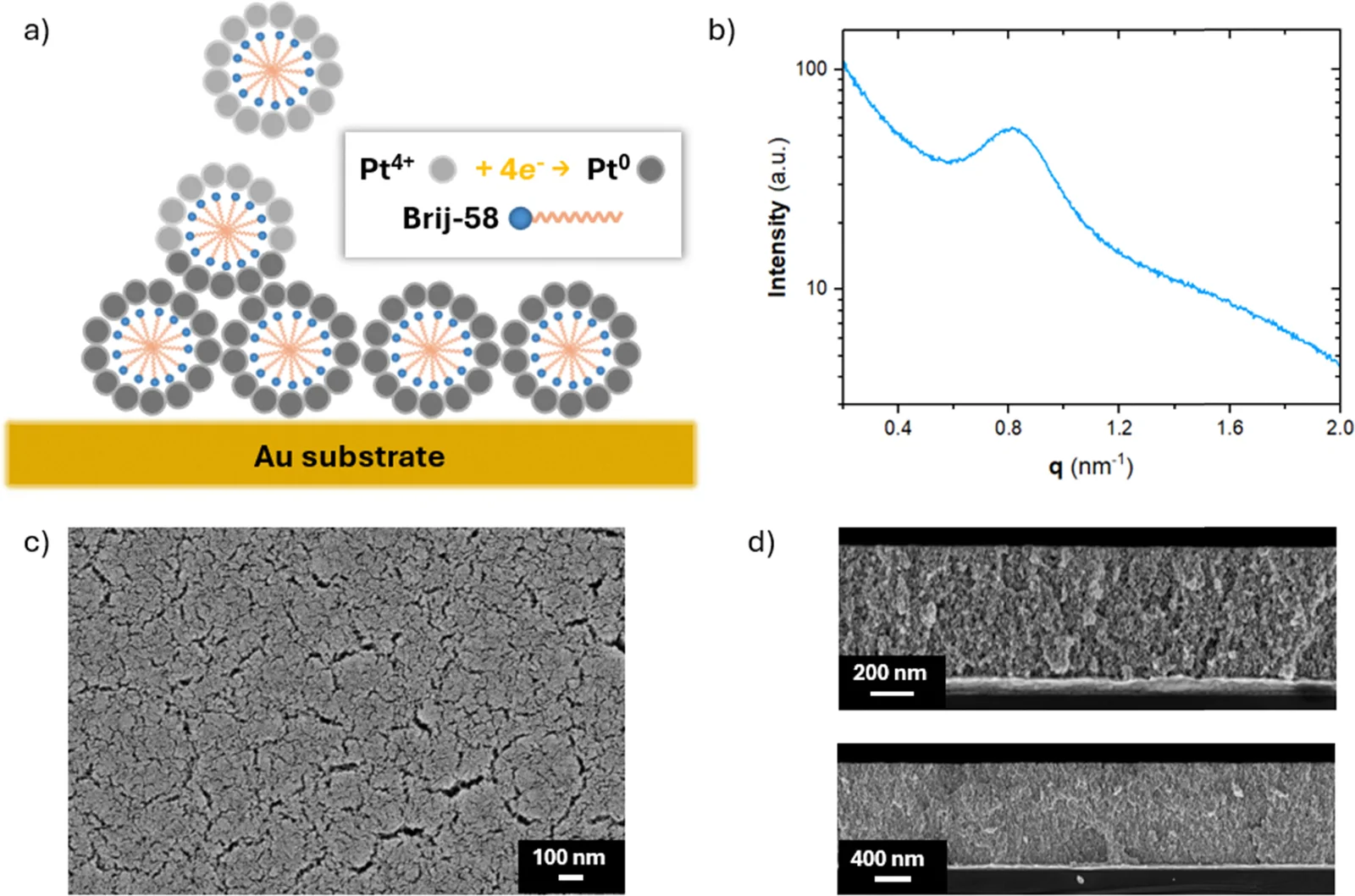
Formation of mesoporous Pt films. (a) Concept of synthesis *via* soft templating process using a non-ionic surfactant (Brij-58). The blue circle represents the hydrophilic head of the surfactants. (b) SAXS profile of a 500 nm-thick Pt film. (d) In-plane and (c) cross-sectional SEM images of mesoporous Pt films.

By tuning the electrodeposition parameters, both the pore structure and thickness of the mesoporous Pt films can be optimized. To identify an appropriate deposition potential, the electroreduction behavior of HCPA was first characterized using linear sweep voltammetry (LSV) (Fig. 4a). A gradual onset of Pt electrodeposition current is observed below 0.5 V_RHE_. At lower potentials, the Pt reduction current is convoluted with features near 0.08 and 0.27 V_RHE_, arising from hydrogen adsorption on the freshly deposited Pt.^33,34^ Below 0 V_RHE_, hydrogen evolution also contributes to the current. This is problematic, as it skews the relation between the current that has passed (charge density) and the total amount of deposited Pt. To stay outside of hydrogen evolution region, and to reduce mass transport limitations induced by high Pt deposition rates that could lead to inhomogeneous deposition, we estimate the practical potential window for deposition to be from 0.15 V_RHE_ to 0.45 V_RHE_. Fig. 4b shows the current during electrodeposition at 0.27 V_RHE_. After an initial sharp drop in current due to local consumption of the HCPA, the current transient stabilized into stable steady-state deposition. To quantify the electrochemically active surface area (ECSA) of the resulting film, cyclic voltammograms were recorded in 0.1 M HClO_4_ (Fig. 4c), and the charge associated with H_upd_ was used as a measure of accessible Pt surface sites. The roughness factor was then defined as the ratio of the ECSA to the geometric electrode area, providing a quantitative descriptor for the surface area amplification induced by the mesoporosity of the film.

**Fig. 4:**
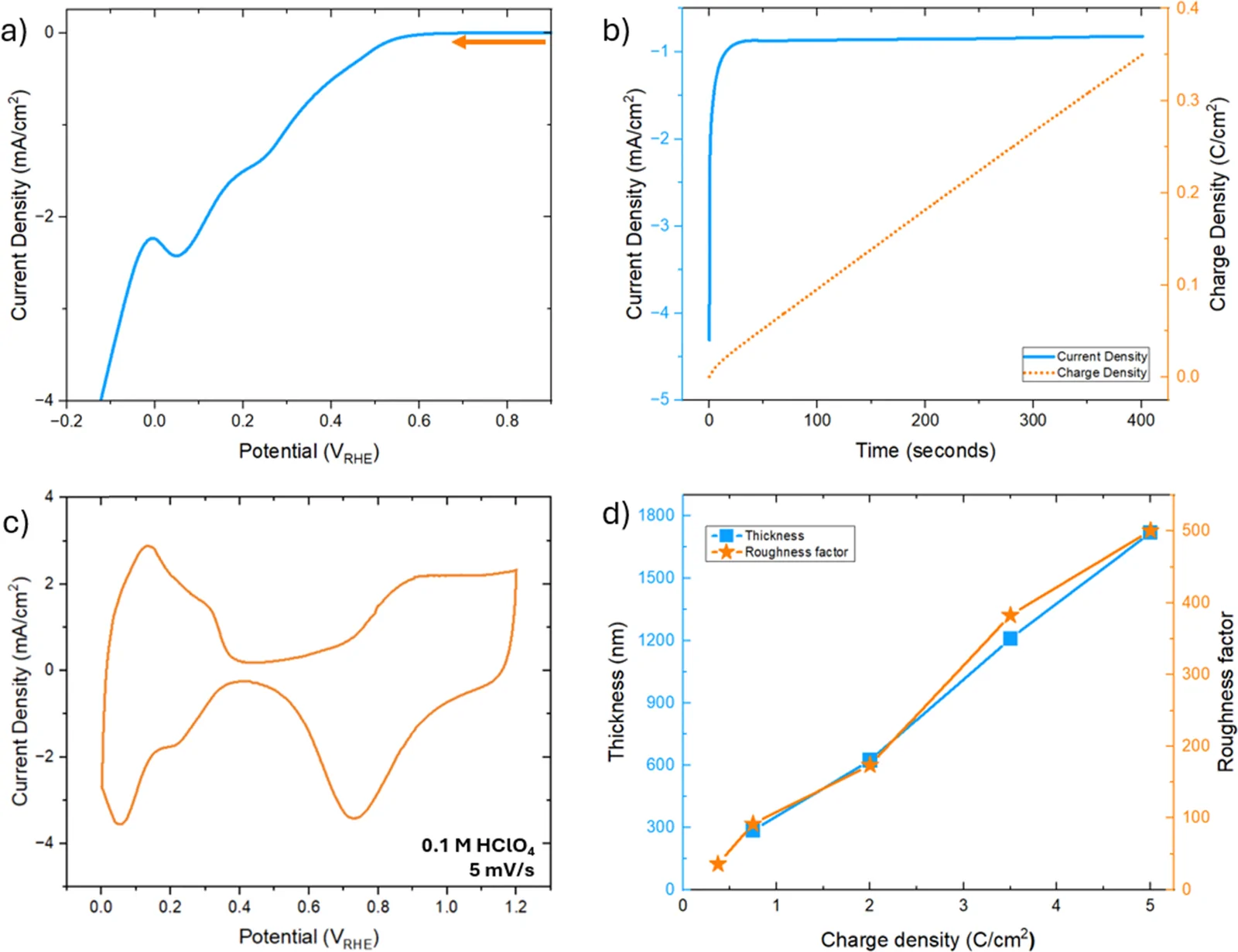
Electrodeposition parameters of mesoporous Pt film preparation. (a) Linear scan voltammetry profile in 0.8 wt% HCPA + 1 wt.% Brij-58, recorded at 5 mV s^−1^. The orange arrow shows the direction of the scan. (b) Current and charge densities during electrodeposition at 0.27 V_RHE_. (c) Typical cyclic voltammogram in 0.1 M HClO_4_ to assess the ECSA of the resulting Pt films. (d) Thickness and roughness factor *vs.* amount of deposited material, expressed in deposition charge density (C cm^−2^). The thickness values were obtained from cross-sectional SEM images.

By varying the deposition time, and thereby the deposition charge density, both film thickness and roughness factor can be controlled (Fig. 4d). The film thickness, determined independently by scanning electron microscopy (SEM), and the roughness factor both exhibit a linear dependence on the deposited charge. This scaling behavior indicates consistent, uniform growth of the porous network during deposition. This means that variation of the deposition time allows tuning of the film thickness, while maintaining a constant pore morphology.

To obtain more detailed insight into the pore morphology of the mesoporous Pt film, we combined SEM data, deposition charge, and roughness factor to calculate the porosity and average pore size, assuming a cylindrical pore geometry:3
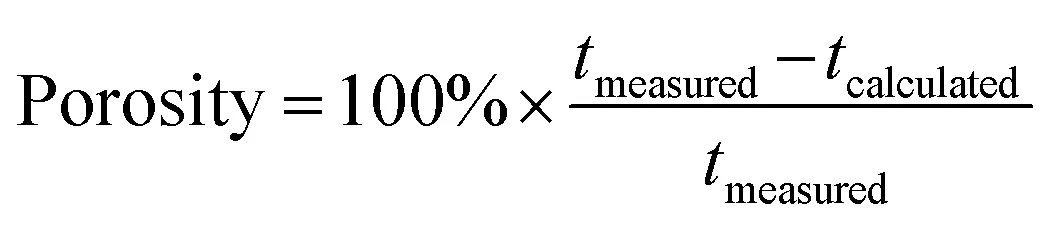


With *t*_measured_ the film thickness measured by SEM and *t*_calculated_ film thickness expected for a non-porous Pt film based on the deposition charge (236 nm per (C cm^−2^)^−1^).4
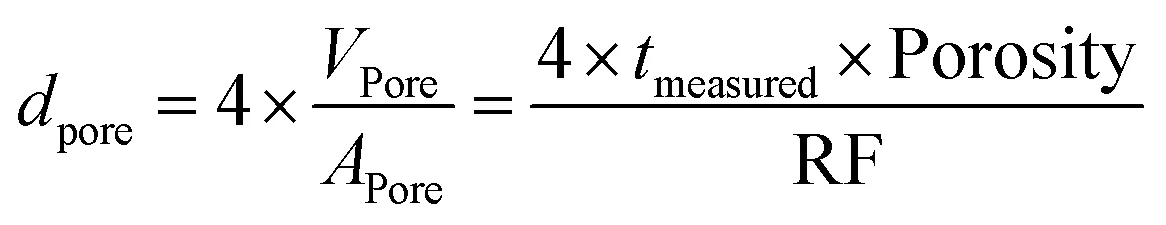


With *d*_pore_, *V*_pore_ and *A*_pore_ the pore diameter, volume and area, respectively, and RF the roughness factor. For the deposition at 0.27 V_RHE_, we find a porosity of approximately 31% and pore size of ∼4 nm.

To vary the pore morphology, the deposition potential can be used. When the potential is lowered below 0.27 V_RHE_, the deposition current increases, which can affect the degree to which the accessible space around the surfactant micelles is fully filled with Pt. Indeed, deposition at slightly lower potential, 0.22 V_RHE_, and correspondingly a larger Pt reduction current (∼1.7 mA cm^−2^) leads to a more open pore structure with a film porosity of ∼50% and pore size of ∼9.7 nm. We attribute this increase in porosity and pore size to “messier” growth at higher current density, where the space around the micelles is filled to a lesser extent. Another contributing factor is the increasing level of hydrogen adsorption on the growing Pt film, which has also been shown to affect the deposition process.^34^

The pore size and porosity that can be tuned through the deposition potential affect the *in situ* XAS measurements in several ways. From the electrochemical point of view, the higher porosity and pore size produced at lower potential is advantageous, as such an open film structure facilitates better mass transport under electrocatalytic conditions and more bulk-like electrolyte behavior. In addition, the porosity affects the probing depth of the XAS measurements. This can be quantified using eqn (1) and (2), considering the X-ray attenuation length calculated using SI Section S1. For our experimental geometry the attenuation length at the Na K-edge is ∼158 nm for the film deposited at 0.27 V_RHE_, whereas it is ∼214 nm for the film deposited at 0.22 V_RHE_. Hence, a thicker film is required to attain full attenuation of the X-rays within the film if it has a more open structure. Finally, the pore size of the film also impacts the degree of interface-sensitivity of the measurements. For example, if we approximate the pores as cylindrical and assume an electrical double layer thickness of 0.5 nm, than 44% of the electrolyte in the film is part of the double layer for the 4 nm pores produced at 0.27 V_RHE_. For the 9.7 nm pores produced at 0.22 V_RHE_, this is 20%. In our proof-of-concept here, we chose to optimize for maximum interface sensitivity. Therefore, we proceeded with films deposited at 0.27 V_RHE_.

To demonstrate how the mesoporous Pt film facilitates soft XAS studies on interfacial ions, we tracked the behavior of Na^+^ ions at the Pt-electrolyte interface in several electrolytes. The mesoporous Pt films deposited on SiN_*x*_ chips were mounted in a polyether ether ketone (PEEK) flow cell using a metal holder that also served as electrical contact. The cyclic voltammogram recorded in the flow cell in 0.1 M NaOH (Fig. 5b) shows the sharp H_upd_ and oxide features typical of clean polycrystalline platinum in alkaline media, confirming the clean, bubble-free assembly of the cell and proper electrical contact. To facilitate alignment of the X-ray beam, Ag paste was applied to the SiN_*x*_ chip to cover all regions except the thin membrane window (see Fig. 5c). The intensity of the Ag M_2,3_-edges (∼603.8 and ∼573.0 eV) provides a reliable marker for locating the membrane window, enabling reproducible alignment of the X-ray beam with the active electrode area. Note that the same can be done in the tender X-ray range using the Ag L-edges. Once aligned, O K-edge spectra can be used to verify that the pore structure is free of surfactant template remnants and fully accessible to the electrolyte (see Fig. S2 in the SI and the corresponding discussion).

**Fig. 5:**
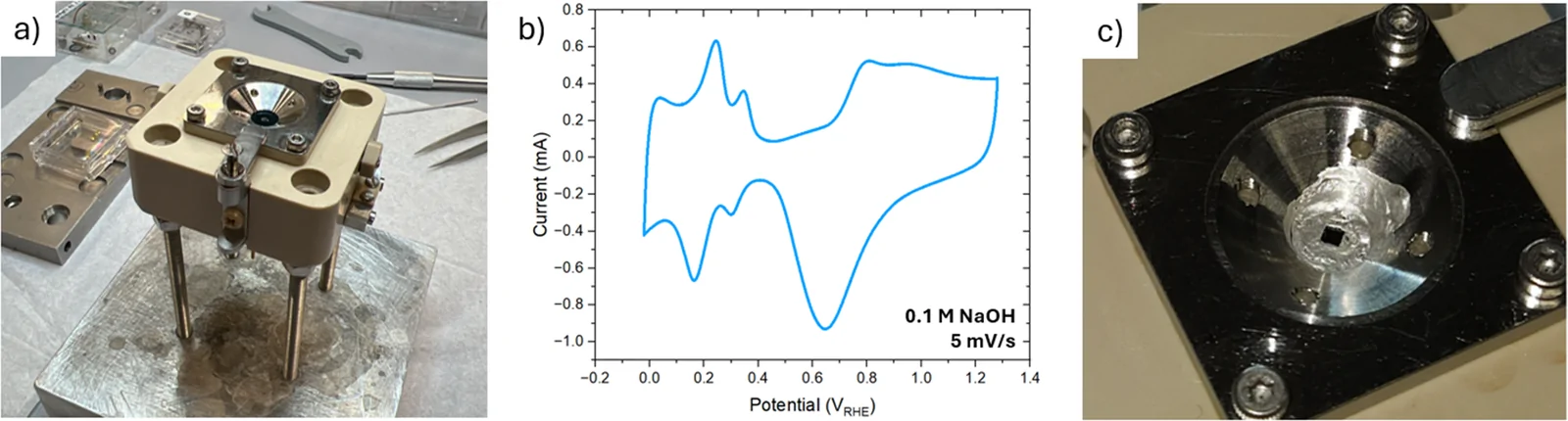
(a) Spectro-electrochemical flow cell at the ISISS beamline at BESSY II. (b) Cyclic voltammetry of a mesoporous Pt film mounted inside the spectro-electrochemical flow cell in 100 mM NaOH. (c) Ag paste applied on the SiN_*x*_ chip exposed in the endstation analysis chamber for the alignment of the window to the incident X-ray beam and to the detector.

Using the Na K-edge as an element-specific probe, Na^+^ ions were monitored in alkaline media at 5 mM NaOH (pH 11.7) and 100 mM NaOH (pH 13.0), as well as in acidic media in 50 mM NaClO_4_ + 50 mM HClO_4_ (pH 1.3). Spectra were recorded for three Pt surface states: the H-covered surface in the H_upd_ region, the OH/H_2_O covered surface in the “double layer” region, and the surface oxide. Importantly, all spectra are recorded with the same sample and a fixed alignment, so that the absolute intensities of the spectra can be compared. These intensities, particularly at the post-edge, are thus a direct measure for the variation in Na^+^ concentration at the Pt-electrolyte interface.

As shown in Fig. 6a and b, the dataset recorded in this manner makes it possible to assess the effects of pH, electrolyte concentration, and surface structure/potential on the interfacial Na^+^ concentration. To interpret these effects, it is common to use the framework of the potential of zero charge (PZC). The PZC is a material-specific property of the electrode surface, which determines at what potential the surface will be charge neutral. At potentials below the PZC, the electrode surface charges negatively and cations are increasingly attracted. Along the same lines, the electrode surface charges positively above the PZC, repelling cations and attracting anions. Thus, the difference between the applied potential and the PZC provides a first indication of the interfacial ion concentration that can be expected.

**Fig. 6:**
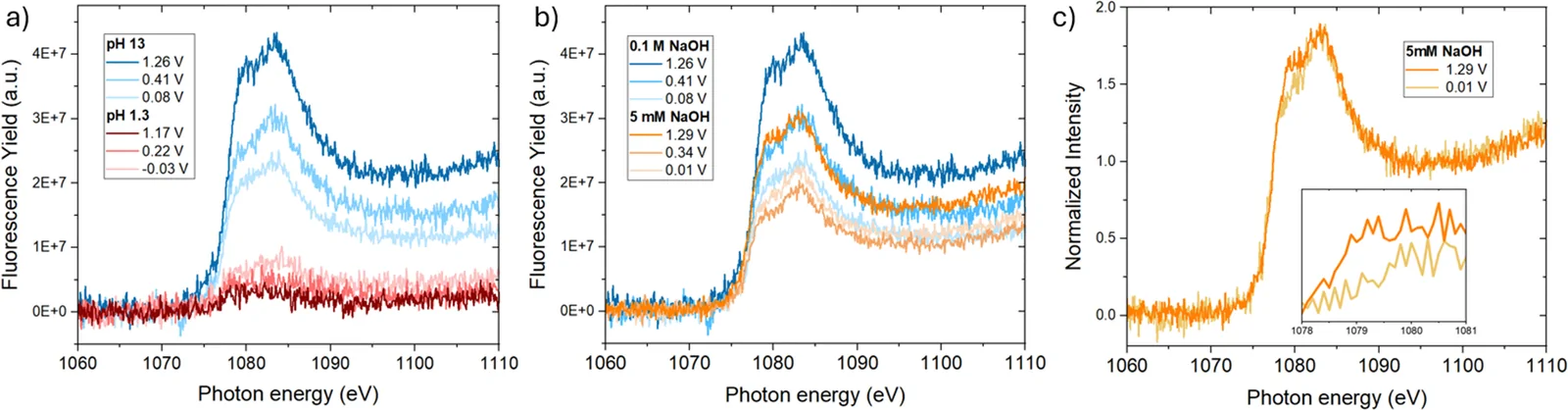
Observation of the interfacial Na^+^ cation concentration and hydration shell *via* Na K-edge XAS. Background-subtracted spectra of (a) alkaline (pH 13) *vs.* acid (pH 1.3) and (b) 100 mM *vs.* 5 mM NaOH, at different applied potentials covering the oxide, double layer, and H_upd_ regions. (c) Normalized spectra of 5 mM NaOH in the oxide (1.29 V) and H_upd_ (0.01 V) regions. Potentials are expressed *vs.* RHE.

We first use the potential *vs.* PZC to analyze the strong pH effect that can be observed in Fig. 6a. When comparing spectra recorded at the same potential *vs.* RHE, it is clear that cations are much more strongly attracted to the Pt surface in pH 13 compared to pH 1.3. The reason for this is that the PZC is a pH-independent property,^35^ whereas the RHE scale is pH-dependent. As a result, potential *vs.* PZC is (13–1.3) × 0.059 = 0.69 V lower in pH 13 compared to pH 1.3, making it much more attractive to cations.

Fig. 6b shows the comparison between 5 mM NaOH and 100 mM NaOH, where again a significant difference in interfacial Na^+^ concentration can be observed. This difference is again in line with expectation, as there is a difference in pH and bulk Na^+^ concentration, both of which steer the interfacial Na^+^ concentration to higher values for the 100 mM NaOH case.

Finally, we can also assess the effect of surface structure/potential on the interfacial Na^+^ concentration. It can be observed that in alkaline electrolytes, the cation concentration rises when increasing the applied potential, against the naïve expectation of the PZC picture. In acidic electrolyte, this is not observed. These observations show that surface chemistry necessitates nuancing of the basic PZC picture, which will be the topic of future studies.

In addition to the interfacial ion concentration quantification, the Na K-edge spectra can also be used to analyze the local hydration environment of the Na^+^ ions. As shown in Fig. 6c, the Na K-edge shows a potential dependent shoulder feature at approximately 1078–1081 eV. Modeling of Na K-edge spectra of solvated Na^+^ ions has shown that the intensity increase in this shoulder can be attributed to the distortion or partial opening of the Na^+^ hydration shell.^36^ Based on this, it is clear that the oxide-covered surface (1.26 V_RHE_) induces more distortion in the hydration shell of the interfacial Na^+^ ions than the hydrogen-covered surface (0.01 V_RHE_). This may indeed be expected, because the Na^+^ concentration at the interface is higher for the oxide covered surface, causing more crowding-induced distortion of the hydration shell. In addition, it may be that the O or OH groups on the oxide-covered surface partially replace water as ligands.

## Conclusion

In summary, we demonstrated an approach for probing ions at the electrode–electrolyte interface under well-defined electrochemical conditions using *in situ* XAS. Central to this methodology is the use of mesoporous Pt films with an exceptionally high surface-area-to-volume ratio, which strongly boosts the interfacial ion signals with respect to those from the bulk. The mesoporous Pt films were fabricated by electrodeposition onto SiN_*x*_ windows *via* a straightforward and well-controlled soft-templating process, yielding homogeneous mesoporous electrodes suitable for *in situ* XAS measurements. Using this approach, we showed that it is possible to track the interfacial Na^+^ concentration and to probe changes in the ion hydration shell. We show how measurements while varying the electrolyte and potential enable studies on the effects of *e.g.* pH, ion concentration, and surface structure/potential on the formation of the electrical double layer. These measurements demonstrate that *in situ* XAS can directly capture ion-specific behavior at electrified interfaces, providing insight into interfacial processes that are difficult to access with conventional electrochemical or spectroscopic techniques. We anticipate that this methodology can be extended to other ionic or molecular species that participate in electrocatalytic and energy storage processes, offering new opportunities to elucidate interfacial phenomena in electrochemical energy conversion and catalysis.

## Conflicts of interest

There are no conflicts to declare.

## Supplementary Material

CP-OLF-D6CP01946C-s001

CP-OLF-D6CP01946C-s002

## Data Availability

The data supporting this article have been included as part of the supplementary information (SI). Supplementary information is available. See DOI: https://doi.org/10.1039/d6cp01946c.
