# Supplementary material for: A mesoporous electrode strategy for probing ions at the electrode–electrolyte interface using in situ X-ray absorption spectroscopy
Source: Phys Chem Chem Phys. 2026 Oct 27. Online ahead of print. doi: 10.1039/d6cp01946c (PMC13726401; doi:10.1039/d6cp01946c)
Supplement: Supplementary file 1 [file CP-OLF-D6CP01946C-s002.pdf]

Supplementary Information (SI) for Physical Chemistry Chemical Physics.
This journal is © the Owner Societies

Supporting information for

A Mesoporous Electrode Strategy for Probing Ions at the Electrode-Electrolyte Interface Using In Situ X-ray Absorption Spectroscopy

Jan Sebastian Dominic Rodriguez[1,2], *Alfred Larsson*[1], *Nipon Deka*[3], *Rianne Joritsma*[1], *Hassan Javed*[1], *Kees Kolmeijer*[1], *Ajsa Cuprija*[4], *Wanli Li*[5], *Nicole Barber*[5], *Adam M. Squires*[5], *Detre Teschner*[6,7], *Aliakbar Ghafari*[6,7], *Cheng-Hao Chuang*[2], *Rik V. Mom*[1,*]

[1]Leiden Institute of Chemistry, Leiden University, Leiden, the Netherlands

[2]Department of Physics, Tamkang University, New Taipei City, Taiwan

[3]Department of Atomic-Scale Dynamics in Light-Energy Conversion, Helmholtz-Zentrum Berlin für Materialien und Energie, Albert-Einstein-Straße 15, 12489 Berlin, Germany

[4]Division of Synchrotron Radiation, Lund University, Lund, Sweden

[5]Department of Chemistry, University of Bath, Bath, BA2 7AY, United Kingdom

[6]Department of Inorganic Chemistry, Fritz-Haber-Institut der Max-Planck-Gesellschaft, Faradayweg 4–6, 14195, Berlin, Germany

[7]Max Planck Institute for Chemical Energy Conversion, Stiftstrasse 34-36, 45470, Mülheim an der Ruhr, Germany

*r.v.mom@lic.leidenuniv.nl

S1 X-ray attenuation length calculations

X-ray attenuation lengths in bulk electrolyte and electrolyte-filled mesoporous Pt films were calculated using the CXRO online calculator, based on the tabulated data of Henke *et al.*[1] For the calculation, the density and atomic composition of the attenuating material are required.

For bulk electrolyte the density was approximated at 1. The composition was calculated as:

$$c_H = \frac{2\rho_{H_2O}}{M_{H_2O}} \qquad (S1)$$

$$c_O = \frac{\rho_{H_2O}}{M_{H_2O}} \qquad (S2)$$

$$c_{Na} = c_{NaOH} \qquad (S3)$$

Where c_i, ρ_i, and M_i are the concentration, density and molar mass of species i, respectively. For example, for a 0.1 M NaOH solution, this leads to a composition of $H_{111.16}O_{55.61}Na_{0.1}$. The density of the electrolyte was approximated as:

$$\square_{electrolyte} = \square_{H_2O} + c_{NaOH}M_{Na} \quad (S4)$$

For the electrolyte-filled mesoporous Pt film, the density and composition were calculated by considering the porosity, $\square$, of the film.

$$c_H = \square\frac{2\square_{H_2O}}{M_{H_2O}} \quad (S5)$$

$$c_O = \square\frac{\square_{H_2O}}{M_{H_2O}} \quad (S6)$$

$$c_{Na} = \square c_{NaOH} \quad (S7)$$

$$c_{Pt} = (1 - \square)\frac{\square_{Pt}}{M_{Pt}} \quad (S8)$$

For a film with 31% porosity and 0.1 M NaOH electrolyte, this leads to a composition of $H_{34.44}O_{17.22}Na_{0.03}Pt_{75.87}$.

The density of the film was calculated as:

$$\square_{film} = \square(\square_{H_2O} + c_{NaOH}M_{Na}) + (1 - \square)\square_{Pt} \quad (S9)$$

For a film with 31% porosity and 0.1 M NaOH electrolyte, this leads to a density of 15.11 g/cm^3.

S2 Cell Assembly and Electrochemical Verification

To ensure reliable electrochemical operation during the operando X-ray absorption spectroscopy (XAS) measurements, the liquid cell and mesoporous Pt electrode were subjected to a series of electrochemical and X-ray checks before and during data acquisition. These checks were designed to quickly identify potential sources of experimental artefacts, including incomplete wetting of the mesoporous film, poor electrical contact, contamination of the cell, residual surfactant in the mesoporous Pt, and partial loss of the Pt film over time (e.g. due to partial delamination). Early detection of such issues is crucial in the in situ experiments, as it prevents the waste of measurement time on a system that is not in a suitable condition for reliable XAS measurements.

The liquid cell was first filled with water to check for major leaks and subsequently filled with electrolyte to check for proper electrochemical contact. Such contact may be absent in case of failing electrical connections, or if bubbles are trapped within the cell (most commonly at the working electrode or at the tip of the reference electrode). These issues can be rapidly identified by 1) the absence of an open circuit potential between the reference and working electrode and between the refence and counter electrodes and/or 2) by a high solution resistance between the reference and working electrode and between the refence and counter electrodes. The solution resistance is measured as the high-frequency impedance between two electrodes. In our system, the typical resistance between reference and working electrodes was 70-80 Ohm.

Cyclic voltammetry was subsequently used to assess the cell/sample cleanliness and electrochemical accessibility of the porous film. Fig. S1 shows typical voltammograms of a clean, stable film obtained in the XAS cell. The cleanliness of the film can be (roughly) assessed by comparing the CV to literature data on polycrystalline Pt in alkaline electrolyte. Extra peaks in the CV, reduced sharpness of the hydrogen adsorption/desorption features (0.1 V_{RHE} to 0.4 V_{RHE}), and reduced sharpness of the Pt oxidation feature at ~0.8 V_{RHE} are typical indicators of the presence of contaminants. In addition, as described in the main text, the roughness factor (i.e. the electrochemically accessible surface area) can be calculated from the current magnitude of the hydrogen adsorption/desorption (H_{UPD}) features. Roughness factors lower than those determined after the film preparation indicate that either the mesoporous Pt film is incompletely wetted by the electrolyte, or that part of the mesoporous film detached from the SiN_x X-ray window. Note that in our experience, several 10s of CV cycles are required to obtain a stable, clean CV. XAS measurements should be conducted only after such stabilization.

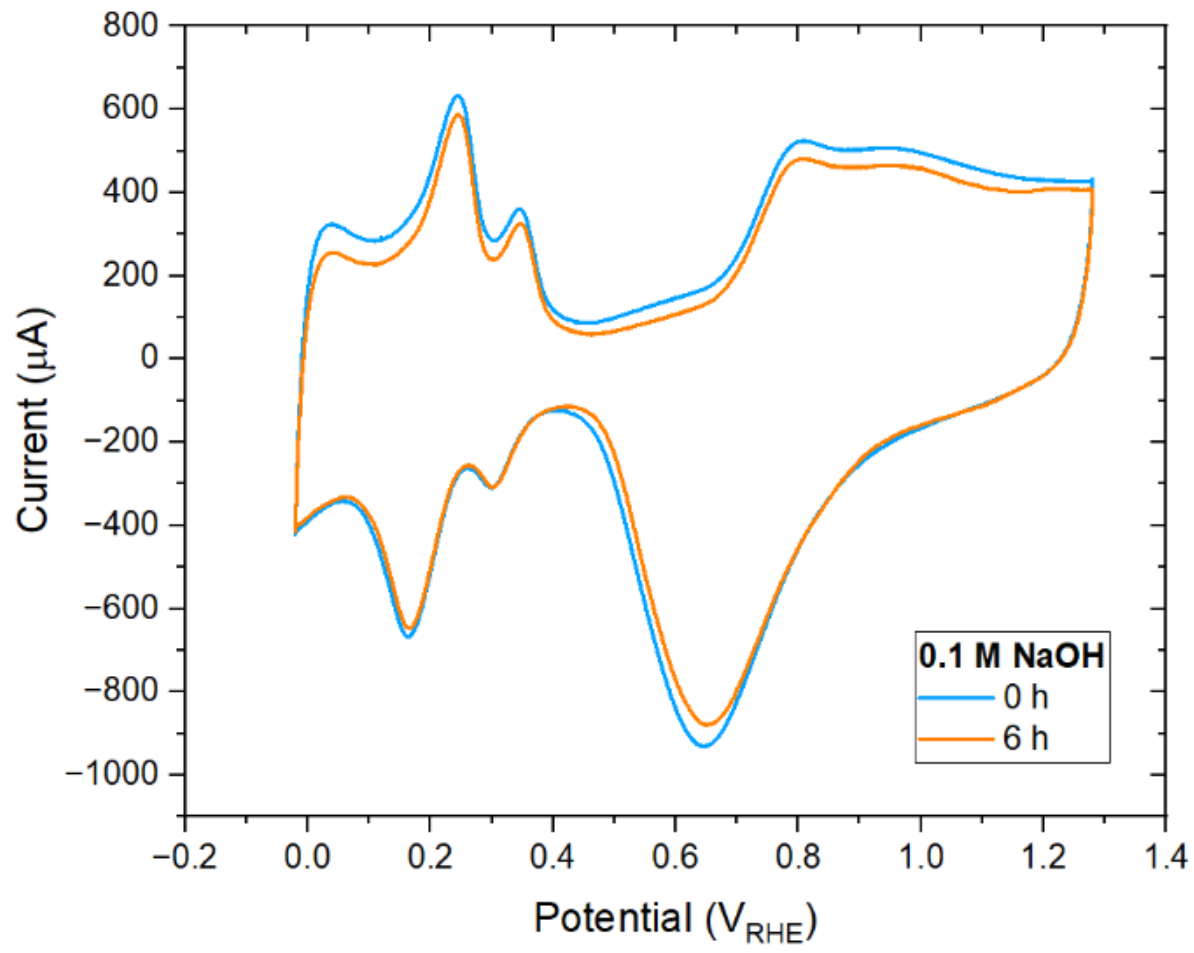

Figure S1. Cyclic voltammograms of the mesoporous Pt electrode recorded in 0.1 M NaOH at the beginning of the XAS experiment and after 6 h. Similar CV responses indicate that the electrochemically accessible surface of the mesoporous Pt remained stable over this period.

To assess the stability of the mesoporous Pt during the XAS measurements, CVs were additionally recorded in the alkaline electrolyte at intervals of approximately 2 hours throughout the experiment. Repeated CV measurements provided a direct check for changes in the electrochemically active surface area that could result from progressive delamination or loss of part of the mesoporous film. Representative CVs are shown in Fig. S1. The stability of the electrochemical response, together with the absence of visible changes in the cell throughout the measurements, was therefore used as evidence that the mesoporous Pt remained electrochemically accessible and mechanically stable during the X-ray measurements.

The quality of the liquid environment and pore wetting was additionally assessed using O K-edge spectra. Figure S2a shows a sample with clear indications of contamination, including a pronounced signal at 531-533 eV that may arise from residual surfactant and/or O₂ in incompletely wetted pores.[2] The strong variation in the water-related signal (535-545 eV) throughout the measurement series further indicates changes in pore wetting, which could result in corresponding variations in the measured Na signal.

In contrast, the spectra in Fig. S2b show a low 531-533 eV contribution at low potential, indicating a clean system. At 1.26 V, a contribution around 531-532 eV appears,[3] consistent with the Pt oxidation expected at

this potential. At higher energies the spectra are dominated by the characteristic features of liquid water, including a shoulder around 535 eV and a broad feature between approximately 537 and 542 eV.[4,5] In principle, these features could be used to analyze the water structure within the pores, and its response to electrode potential. However, since the bulk water contribution from the non-EDL parts of the pores is likely to dominate the signal, and the spectra are relatively noisy due to the low X-ray penetration depth in the O K-edge energy range, the data is of insufficient quality for such an analysis. Instead, we only use the essentially constant intensity of the water signal as an indicator for constant wetting of the mesoporous film.

Finally, Na K-edge spectra were used to verify the presence and reproducibility of the Na-containing electrolyte signal relevant to the measurements presented in the main text. Quick scans at the Na K-edge were similarly used to confirm a reproducible X-ray absorption signal before acquisition of the detailed spectra. Together with the electrochemical checks, these measurements provided complementary verification of the cell filling, electrical connection, electrochemical stability, and X-ray signal quality before and during the operando measurements.

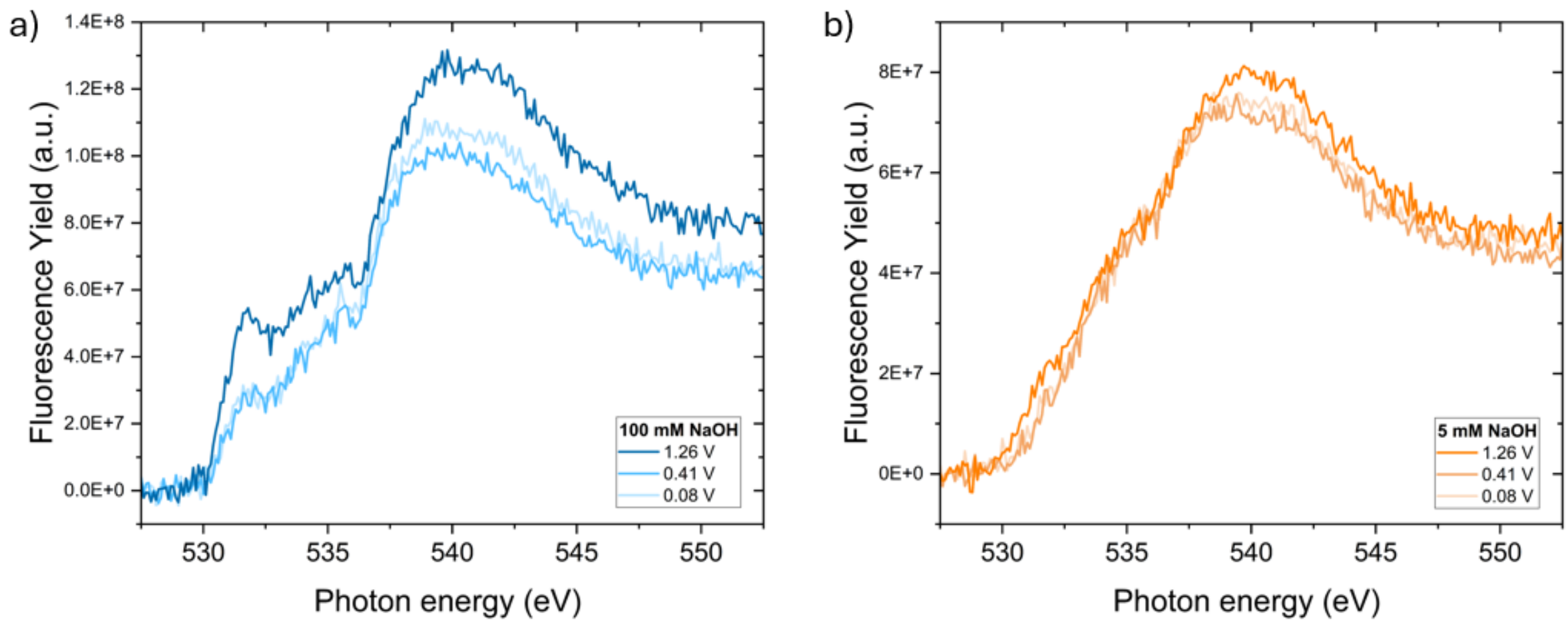

Figure S2. In situ O K-edge spectra of mesoporous Pt in NaOH electrolyte (a) Example of a contaminated sample with non-constant wetting of the pores. (b) Example of clean, electrochemically responsive sample with constant pore wetting.

S3 XAS data treatment

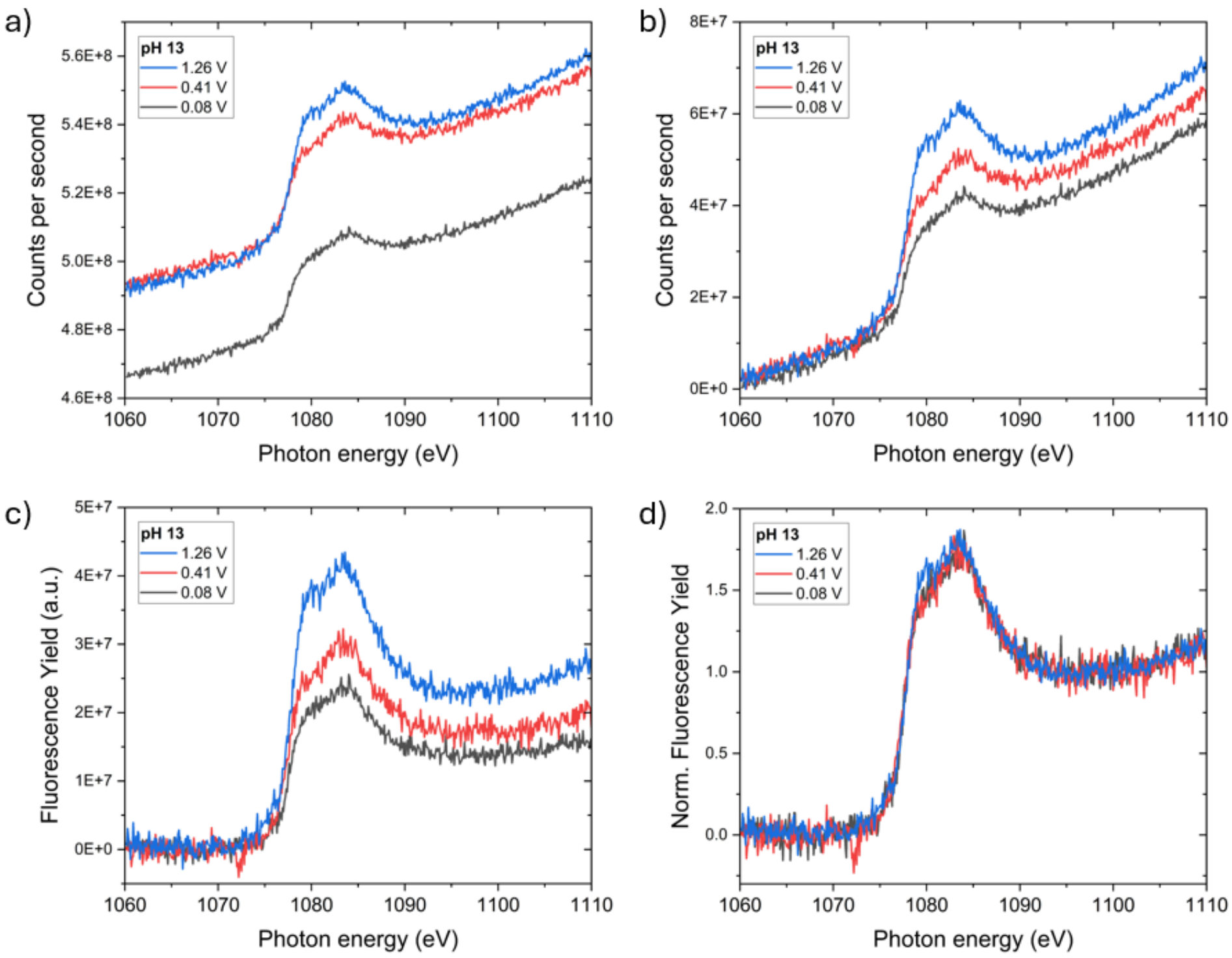

Figure S3. Data processing of XA spectra for determination of edge jump magnitude and normalization. a) Raw data recorded from the beam line. b) Spectra after subtracting the minimum value. c) Background-subtracted data. d) Normalized spectra. The normalization range was set to 1095-1100 eV. The results in S1c are presented in Fig. 6a and 6b in the main text.

As shown in Fig. S3a, the raw fluorescence-yield spectra contain a substantial background signal and a somewhat energy-dependent baseline. Data treatment is therefore required to isolate the absorption-edge response and obtain a consistent measure of the Na⁺ signal.

The spectra were processed in three steps: (i) minimum-value subtraction, (ii) pre- and post-edge background subtraction, and (iii) normalization. The resulting spectra are shown in Fig. S3b–d.

i. **Minimum-value subtraction.** The minimum value of each spectrum was first subtracted from all data points. This step sets the lowest intensity in the spectrum to zero and removes the arbitrary offset associated with the fluorescence count rate (which is influenced by, amongst others, stray visible light in the measurement chamber). Importantly, subtracting a constant does not change the magnitude of the edge jump or the relative spectral shape. This provides a common baseline for the subsequent background subtraction and allows the edge-jump magnitude to be determined consistently.

ii. **Background subtraction.** The minimum-subtracted spectra were then corrected for the energy-dependent background using linear pre- and post-edge fits. The pre-edge fit was extrapolated to the edge energy, E_0, and the post-edge fit was used to remove the residual energy dependence above the edge. The background-subtracted spectra are shown in Fig. S3b. The fitted slopes of the pre- and post-edge were applied consistently throughout each set of measurements.

The complete background-subtraction procedure is described by:

$$y = \begin{cases} x < E_o, & y_{sub} - m_{pre}(x - x_{min}) + c \\ x > E_o, & y_{sub} - m_{pre}(x - x_{min}) - (m_{post} - m_{pre})(x - E_o) + c \end{cases} \quad \text{(S10)}$$

where x is the photon energy, E_o is the edge energy, y_{sub} is the minimum-subtracted intensity, x_{min} is the starting photon energy of the spectrum, m_{pre} and m_{post} are the slopes of the pre- and post-edge lines, respectively, and c is the offset applied as a secondary correction to account for noise sensitivity in the minimum-value subtraction described in step (i).

iii. **Normalization.** To compare spectral shapes, as in Fig. 6b, the spectra were normalized by the edge-jump magnitude, defined as the difference between the average intensity in the post-edge range (1095–1100 eV) and the pre-edge background. In simple terms, the background-subtracted data were divided by the average background-subtracted intensity in the post-edge range. This removes the influence of the overall intensity scale and allows the spectral shapes to be compared directly. The normalized spectra are shown in Fig. S3c and d.

References

(1) Henke, B. L.; Gullikson, E. M.; Davis, J. C. X-Ray Interactions: Photoabsorption, Scattering, Transmission, and Reflection at *E* = 50-30,000 eV, *Z* = 1-92. *Atomic Data and Nuclear Data Tables* **1993**, *54* (2), 181–342. https://doi.org/10.1006/adnd.1993.1013

(2) Wurth, W.; Stöhr, J.; Feulner, P.; Pan, X.; Bauchspiess, K. R.; Baba, Y.; Hudel, E.; Rocker, G.; Menzel, D. Bonding, Structure, and Magnetism of Physisorbed and Chemisorbed O 2 on Pt(111). *Phys. Rev. Lett.* **1990**, *65* (19), 2426–2429. https://doi.org/10.1103/PhysRevLett.65.2426

(3) Miller, D. J.; Öberg, H.; Kaya, S.; Sanchez Casalongue, H.; Friebel, D.; Anniyev, T.; Ogasawara, H.; Bluhm, H.; Pettersson, L. G. M.; Nilsson, A. Oxidation of Pt(111) under Near-Ambient Conditions. *Phys. Rev. Lett.* **2011**, *107* (19), 195502. https://doi.org/10.1103/PhysRevLett.107.195502

(4) Sellberg, J. A.; Kaya, S.; Segtnan, V. H.; Chen, C.; Tyliszczak, T.; Ogasawara, H.; Nordlund, D.; Pettersson, L. G. M.; Nilsson, A. Comparison of X-Ray Absorption Spectra between Water and Ice: New Ice Data with Low Pre-Edge Absorption Cross-Section. *J. Chem. Phys.* **2014**, *141* (3), 034507. https://doi.org/10.1063/1.4890035

(5) Wernet, Ph.; Nordlund, D.; Bergmann, U.; Cavalleri, M.; Odelius, M.; Ogasawara, H.; Näslund, L. Å.; Hirsch, T. K.; Ojamäe, L.; Glatzel, P.; Pettersson, L. G. M.; Nilsson, A. The Structure of the First Coordination Shell in Liquid Water. *Science* **2004**, *304* (5673), 995–999. https://doi.org/10.1126/science.1096205
